# Exploring the Role of ChatGPT in Cardiology: A Systematic Review of the Current Literature

**DOI:** 10.7759/cureus.58936

**Published:** 2024-04-24

**Authors:** Aditi Sharma, Tejas Medapalli, Michaella Alexandrou, Emmanouil Brilakis, Anand Prasad

**Affiliations:** 1 Department of Medicine, Division of Cardiology, University of Texas (UT) Health San Antonio, San Antonio, USA; 2 Department of Cardiology, Minneapolis Heart Institute, Minneapolis, USA

**Keywords:** cardiovascular care, patient education, language models, artificial intelligence, cardiology, chatgpt

## Abstract

Chat Generative Pre-Trained Transformer (ChatGPT) is a chatbot based on a large language model that has gained public interest since its release in November 2022. This systematic review examines the current literature on the potential applications of ChatGPT in cardiology.

A systematic literature search was conducted to retrieve all publications on ChatGPT in PubMed, Scopus, MedRxiv, and the Cochrane Library published on or before September 30, 2023. Search terms relating to ChatGPT and cardiology were used. Publications without relevance to ChatGPT and cardiology were excluded. The included publications were divided into cohorts. Cohort A examined ChatGPT’s role in improving patient health literacy. Cohort B explored ChatGPT’s role in clinical care. Cohort C examined ChatGPT’s role in future literature and research. Cohort D included case reports that used ChatGPT.

A total of 115 publications were found across all databases. Twenty-four publications met the inclusion criteria and were included in the review. Cohort A-C included a total of 14 records comprised of editorials/letters to the editor (29%), research letters/correspondence (21%), review papers (21%), observational studies (7%), research studies (7%), and short reports (7%). Cohort D included 10 case reports. No relevant systematic literature reviews, meta-analyses, or randomized controlled trials were identified in the search.

Based on this review of the literature, ChatGPT has the potential to enhance patient education, support clinicians providing clinical care, and enhance the development of future literature. However, further studies are needed to understand the potential applications of ChatGPT in cardiology and to address ethical concerns regarding the delivery of medical advice and the authoring of manuscripts.

## Introduction and background

Artificial intelligence (AI) has opened new possibilities in healthcare. Natural language processing is a field within AI focusing on computers and human language. Large language models are an advancement within the field of natural language processing and are designed to analyze large amounts of information using neural network learning [1]. In November 2022, Open AI (San Francisco, CA) launched Chat Generative Pre-Trained Transformer (ChatGPT) to the public [2]. Unlike other databases such as Google (Mountain View, CA) or Microsoft Bing (Redmond, WA), ChatGPT generates answers by searching a pre-uploaded database. This database was developed using approximately 570 gigabytes of information derived from Common Crawl, WebText2, Books1, Books2, and Wikipedia [2,3]. The platform was then fine-tuned by researchers, who tested various prompts and provided feedback. The conversational features of the database raise interest in its potential applications [4]. The most recent free publicly available version of ChatGPT is GPT-3.5, which includes a blend of text and code from before Q4 2021 [2]. ChatGPT-4.0 is a paid version of the platform that has more advanced reasoning capability, can accept and generate visual images, and can be used for creative tasks with content included until April 2023 [5].

The potential applications of ChatGPT have interested healthcare researchers in multiple fields. Since its development, ChatGPT has been demonstrated to correctly answer 60% of United States Medical Licensing Examination questions accurately at par or near passing thresholds, assist in writing medical case reports, and support the development of patient health education materials [6-8]. Over time, various applications of other AI technologies have also been used in cardiology. Some examples of AI implementation in echocardiography include obtaining key measurements for segmentation of heart chamber sizes, estimating left ventricular (LV) ejection fraction, calculating longitudinal strain, and aiding in the identification of valvular disease [9-11]. AI technologies have also been employed in cardiac computed tomography (CT) to automatically calculate coronary artery calcium scores and classify coronary plaques [11,12]. Similar technologies have also been applied to supplement the estimation of ejection fraction and support the quantification of LV mass and scar volume by cardiac magnetic resonance imaging [11].

Currently, limited information exists regarding the potential role and applications of ChatGPT within cardiology. This literature review aims to synthesize current publications and provide commentary on the potential applications and limitations of using ChatGPT in cardiology.

Methods

General Approach

This paper provides a review on ChatGPT 3.5 in Cardiology with goals to identify papers related to the topic. A systematic literature search was conducted to retrieve all English records in PubMed, Scopus, MedRxiv, and the Cochrane Library published before September 30, 2023. Search terms included “ChatGPT” and “Cardiology,” “ChatGPT” and “Heart,” “ChatGPT” and “Cardiac,” and “ChatGPT” and “Cardiovascular”. After obtaining a list of publications, titles were screened to evaluate for their relevance to ChatGPT and cardiology. Two reviewers (A.S. and T.M.) performed study selection and analysis. The two reviewers calibrated the reviews on a shared Excel sheet. The key findings for each article were synthesized using a shared document. Selected studies to be included were compiled together in a shared document. Both reviewers independently analyzed these papers. Subsequently, each independent analysis was synthesized into this review. The final included publications were subdivided into four different cohorts, as detailed below.

Inclusion and Exclusion Criteria

Records were considered for initial review if they were found to be relevant to the role of ChatGPT and cardiology. Articles published from November 2022 to September 2023 were included in this literature review. Specifically, those with relevance to health literacy, clinical care, applications in future literature and research in cardiology were included. Case reports using ChatGPT were also included in the review. The primary objective was to summarize ChatGPT’s potential applications within cardiology.

Case reports and publications that did not mention ChatGPT were excluded. Pre-prints, abstracts, posters, non-English, and duplicate records were also excluded from the analysis.

Objectives and cohorts

A data extraction protocol was employed based on the study objectives and selection criteria. The primary objective of the paper was to summarize ChatGPT’s potential applications within cardiology. The focus of this paper was to identify publications with relevance to four aspects of ChatGPT's role in cardiology. Cohort A included publications that examined ChatGPT’s role in improving patient health literacy. Publications included in cohort B explored ChatGPT’s role in clinical care. Cohort C included articles that examined ChatGPT’s applications in future literature and research. Cohort D included case reports that used ChatGPT. The protocol described above was applied to all cohorts.

## Review

Results

**Figure 1:**
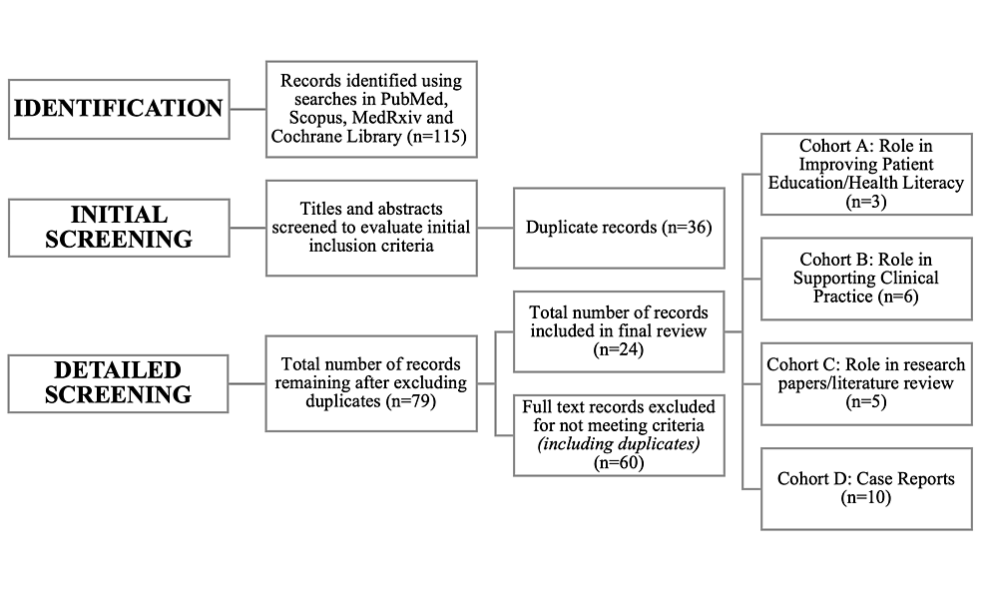
Illustrates the results of the database searches and distribution of articles included in the literature review. Note: This image is the author's own creation.

Initially, a total of 115 records were found across all databases. After the initial screening, 36 duplicate publications were excluded from the study. The remaining publications were screened for relevance, after which 24 articles were included in the final review (Figure 1). Due to the limited number of articles, we were unable to carry out analysis per the preferred reporting items for systematic reviews and meta-analyses (PRISMA) protocol or conduct statistical analyses. The included publications were divided based on their relevance to the identified cohort topics, with descriptive information detailed in Table 1. 

**Table 1:** Descriptive data of publications included in the literature review after screening and application of inclusion criteria.

Descriptive data of publications included in the literature review		
Total number of publications included	n=24	
	Records included in Cohort A-C (n=14)	Records included in Cohort D (n=10)
Continent of publication (based on first author)		
North America	7% (n=1)	80% (n=8)
Europe	57% (n=8)	0% (n=0)
Asia	29% (n=4)	20% (n=2)
Australia	7% (n=1)	0% (n=0)
Type of publication by %		
Editorials/letters to the editor	36% (n=5)	NA
Observational studies	7% (n=1)	
Research letters/correspondence	21% (n=3)	
Research studies	7% (n=1)	
Review papers	21% (n=3)	
Short reports	7% (n=1)	

**Figure 2:**
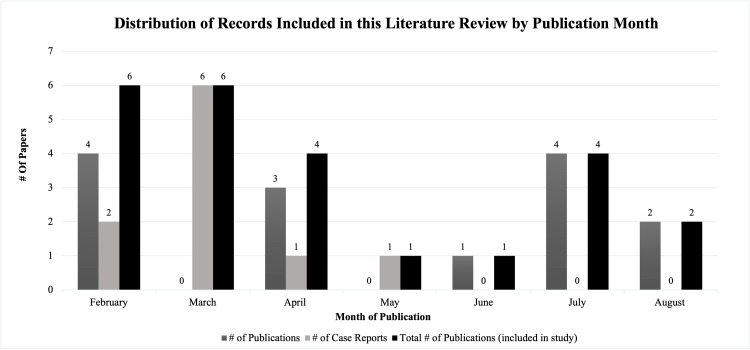
Displays the date of study publication for all publications included in this literature review overtime. Study publication dates ranged from February 2023 to August 2023. Note: This image is the author's own creation.

Publication dates for the included papers ranged from February 2023 to August 2023. These dates were mapped over time, with most research papers noted to be published in February and July 2023 and most case reports published in March 2023, as depicted in Figure 2. No relevant systematic literature reviews, meta-analyses, or randomized controlled trials were identified.

A total of three publications were included in Cohort A, six publications in Cohort B, five publications in Cohort C, and 10 publications in Cohort D. Cohort A-C included a total of 14 records: editorials/letters to the editor (36%), research letters/correspondence (21%), review papers (21%), observational studies (7%), research studies (7%), and short reports (7%). Some case report authors also noted the limitations of using ChatGPT in their publications. These included factually inaccurate information (20%) and inaccurate references (50%). Forty percent of case reports did not comment on the limitations of using the platform. The articles included in this review were analyzed and interpreted with summaries provided in Tables 2, 3, and further discussed below.

**Table 2:** Cohort A-C publications were included in the literature review after screening and application of inclusion criteria with brief summaries. *Country based on affiliation of the first author of the publication. CPR: cardiopulmonary resuscitation.

Cohort	Date published	Country*		Authors	Version of ChatGPT used	Article title	Brief summary
Cohort A	February 2023	South Korea		Ahn [13]	Unknown	Exploring ChatGPT for information of cardiopulmonary resuscitation	Pass examined responses provided by ChatGPT to questions relating to CPR
Cohort A	April 2023	Belgium		Van Bulck et al. [14]	Unknown	What if your patient switches from Dr. Google to Dr. ChatGPT? A vignette-based survey of the trustworthiness, value, and danger of ChatGPT-generated responses to health questions	Compared responses to questions provided by ChatGPT and Google on common cardiovascular diseases
Cohort A	July 2023	United States		Krittanawong et al. [15]	Unknown	Assessing the potential of ChatGPT for patient education in cardiac catheterization care	Asked questions relating to cardiac catheterization and compared responses provided by Google and ChatGPT
Cohort B	July 2023	Netherlands		Gala et al. [16]	ChatGPT-4	The utility of language models in cardiology: a narrative review of the benefits and concerns of ChatGPT-4	Explained how language modeling software works and provided details on the potential uses of ChatGPT for the medical field. Commented on the pros and cons of implementing ChatGPT in healthcare
Cohort B	February 2023	Belgium		Fijacko et al. [17]	Unknown	Can ChatGPT pass the life support exams without entering the American Heart Association course?	Compared responses provided by ChatGPT when asked BLS and ACLS questions
Cohort B	April 2023	Switzerland		Skalidis et al. [18]	Unknown	ChatGPT takes on the European Exam in Core Cardiology: an artificial intelligence success story?	Examined ChatGPT’s ability to European Cardiology exam questions
Cohort B	June 2023	Japan		Kusunose et al. [19]	Unknown	Evaluation of the accuracy of ChatGPT in answering clinical questions on the Japanese Society of Hypertension Guideline	Evaluated ChatGPT responses to Japanese Hypertension guidelines
Cohort B	July 2023	UK		Williams et al. [20]	ChatGPT-3	How will artificial intelligence transform cardiovascular computed tomography? A conversation with an AI model	Asked ChatGPT regarding its potential role in cardiac CT and evaluated responses provided
Cohort B	August 2023	Pakistan		Rizwan et al. [21]	ChatGPT-4	The use of AI in diagnosing diseases and providing management plans: a consultation on cardiovascular disorders with ChatGPT	Examined responses provided by ChatGPT when asked about treatment and management plans for 10 common cardiovascular conditions
Cohort C	February 2023	Belgium		Moons et al. [22]	Unknown	ChatGPT: can artificial intelligence language models be of value for cardiovascular nurses and allied health professionals	Explored ChatGPT responses to a prompt asking the platform to comment on potential applications of the ChatGPT for cardiovascular nursing
Cohort C	February 2023	France		Marchandot et al. [23]	Unknown	ChatGPT: the next frontier in academic writing for cardiologists or a pandora’s box of ethical dilemmas	Review of limitations and possibilities of dilemmas that cardiologists may face with the increasing use of ChatGPT in literature
Cohort C	April 2023	Japan		Nakaya et al. [24]	Unknown	ChatGPT’s ability to classify virtual reality studies in cardiology	Evaluated ChatGPT’s ability to classify virtual reality studies for a literature review
Cohort C	July 2023	Greece		Kleebayoon et al. [25]	Unknown	Does the long-term administration of proton pump inhibitors increase the risk of adverse cardiovascular outcomes? A ChatGPT-powered umbrella review	Literature review article that implemented support from ChatGPT to help with each step of the process
Cohort C	August 2023	Australia		Bart et al. [26]	Unknown	Emerging roles of artificial intelligence (AI) in cardiology: benefits and barriers in a brave new world	Examined how to ethically use ChatGPT to write papers, also looked at current guidelines of various journals

**Table 3:** Case reports included in the literature review with brief summaries.

Date published	Authors	Case report title	Use of ChatGPT (based on text provided in case report)	Limitations noted	Prompts used (based on figures included in case report)
February 2023	Akhter et al. [27]	Acute Pulmonary Edema After Hyperbaric Oxygen Treatment: A Case Report Written with ChatGPT Assistance	Introduction	Inaccurate Reference Citations	"Provide a brief overview of what oxygen therapy is and common indications. This is for my case report."
February 2023	Saeed et al. [28]	Pacemaker Malfunction in a Patient with Congestive Heart Failure and Hypertension	Not reported	Not reported	Not listed
March 2023	Bawa et al. [29]	Appendix Playing Hide and Seek: A Variation to Amyand's Hernia	Introduction, Discussion	Inaccurate Reference Citations	"Write a detailed discussion with references for the same", "Complications of untreated Amyand’s hernia a brief summary with references."
March 2023	Brown et al. [30]	Breaking Bias: The Role of Artificial Intelligence in Improving Clinical Decision-Making	Title, draft sections of the report, and provide critical feedback	Not reported	"What would be a catchy title for a journal article about how AI can help clinicians avoid human bias and errors,” "I'm writing a light piece for a medical journal about the use of AI to help clinicians avoid falling into the traps of human cognitive biases. What are key pitfalls for clinicians to be aware of when using clinical AI."
March 2023	Kattoor et al. [31]	A Case of Unintentional Release of the Watchman FLX Device During Implantation: A Cautionary Tale	Suggested an appropriate title, introduction, abstract, and conclusion	Inaccurate Reference Citations	"Create a case report of …", "Suggest a title for the case report", "Write an abstract in 250 words for the case report", "Write a conclusion for this case report in five sentences", "Write a paragraph about common complications of .. and provide citation"
March 2023	Lal Vallath et al. [32]	Ventricular Septal Rupture and Artificial Intelligence (AI)-Assisted Healthcare.	Collect precise data, conduct a literature review, and correct grammar	Inaccurate Reference Citations	"If I give you links of a few case reports from PubMed can you help me draft an introduction to a case report” "For introduction, please mention these points…” "Can you add points about..".
March 2023	Schussler et al. [33]	Extreme Hyperthermia Due to Methamphetamine Toxicity Presenting As ST-Elevation Myocardial Infarction on EKG: A Case Report Written with ChatGPT Assistance.	Introduction, Abstract	Factually inaccurate information	"Write an abstract in the style of a medical paper describing a case of .. "Write an introductory paragraph in the style of a medical paper describing a case report of …” "Write a paragraph describing an acknowledgment of this medical article written largely with the help of ChatGPT".
March 2023	Raxwal et al. [34]	A Collaborative Case Report Utilizing ChatGPT AI Technology of Traumatic Right Coronary Artery Dissection Resulting in Inferior Wall ST-Elevation Myocardial Infarction	Title, abstract, introduction, and conclusion	Inaccurate Reference Citations, Factually inaccurate information	"Generate 10 keywords for this case report"
April 2023	Afzal et al. [35]	Transient Ischemic Attack in a Patient with Poland Syndrome with Dextrocardia	Writing of clinical case presentation and epidemiology	Not reported	"What is the clinical presentation for a patient with …” "Epidemiology of …” "Dextrocardia in Poland Syndrome"
May 2023	Yazdi et al. [36]	Seizure-Induced Cardiomyopathy: A Case of Takotsubo Cardiomyopathy Following an Epileptic Event	Utilized AI in drafting the manuscript	Not reported	Prompts not revealed in paper, authors input a patient case and asked ChatGPT to write the case using Cureus guidelines

Discussion

This systematic review examines potential applications of ChatGPT in cardiology using a protocol to identify, screen, and interpret currently available literature. The review focused on publications relevant to three potential areas of application of ChatGPT in cardiology (Cohort A-C) and case reports written by ChatGPT (Cohort D). The findings and key comments for each cohort’s publications are detailed in the discussion below. 

Cohort A: Role in improving patient health education and literacy

Health literacy and patient education are important elements of clinical care. Health education materials can often be challenging for patients to understand. As a result, patients may search the internet for answers regarding their medical conditions. ChatGPT’s accessibility suggests a potential role in answering patients’ medical queries and effecting patient health literacy.

Current online sources of patient information include search engines (Google, Yahoo, and Microsoft Bing) and medicine-focused sites such as WebMD. Van Bulck et al. (2023) evaluated ChatGPT responses to four patient questions relating to congenital heart disease, atrial fibrillation, heart failure, and hyperlipidemia. Sample questions included, “What treatment is given to patients with atrial fibrillation?”, “How to lower cholesterol?” and “I am a heart failure patient, what should I do when I am very thirsty?”. A team of 20 experts (19 nurses and one dietician) graded responses provided by ChatGPT and Google Search for trustworthiness, value, and potential danger. Many experts (40%) considered ChatGPT’s answers more valuable than those provided by the Google search engine, while 45% graded them equally valuable. Others observed that the answers provided were too general or incomplete [14]. ChatGPT’s ability to rapidly search information and respond to customized prompts raises interest in its potential ability to support direct patient care. While this skill is similar to features offered by other platforms like Google, Yahoo, and Microsoft Bing. ChatGPT pulls information from a database of information up to a certain date (for ChatGPT 3.5) and synthesizes these points together. In contrast, Google, Yahoo, and Microsoft Bing search all available websites for information and instead provide relevant links for information.

Another aspect of cardiac care that requires patient education is invasive procedures. For example, while cardiac catheterizations are commonly performed, many patients may have a limited understanding of what these procedures entail. ​An exploratory study compared responses by Google search with those from ChatGPT. A total of 18 questions relating to coronary artery disease and general advice for catheterization were asked to both search platforms and graded by cardiologists as “not reliable”, “reliable without explanation”, “reliable”, or “reliable but needs explanation”. Some examples of questions included “What are the symptoms of a heart attack?”, “How do I prepare for a cardiac catheterization?”, and “When should I call my doctor or nurse after a cardiac catheterization?”. Notably, ChatGPT’s answers were graded as more reliable without explanation compared with the answers provided by the Google search engine (33% vs 6%) [15].

Ahn (2023) examined responses given by ChatGPT to questions from patients relating to cardiopulmonary resuscitation (CPR). The author theorized that individuals without basic life support education may have more difficulty understanding CPR guidelines, and as a result, the accessibility of ChatGPT may be beneficial to increasing understanding. The following two questions were asked on the platform, “When I meet a cardiac arrest patient at sea, tell me what to do as a lay rescuer?” and “In addition, is there any difference in the CPR method in cases of cardiac arrest by drowning compared to normal cases?”. These were followed by asking the platform for a source for each of the answers provided. Responses provided by ChatGPT appeared consistent with current publicly available guidelines. When asked to provide sources, the platform stated, “The American Heart Association is a reliable source for information on CPR for lay rescuers. For information on CPR in case of cardiac arrest by drowning, you can refer to the guidelines published by the American Red Cross.” The author observed that compared to trying to comprehend pages of CPR guidelines, it may be easier for the general population to use chat-style programs [13].

The studies above provide insight into ChatGPT’s potential to increase the accessibility of accurate medical information for the general population. The constant accessibility that AI offers can allow patients to get their questions answered at their own pace. However, ChatGPT also comes with limitations, such as the potential inaccuracies that may pose risks to patients and the general population seeking medical knowledge through a generalized platform with limited ability to provide the most up-to-date information. Therefore, further studies are necessary to assess the limitations and future applications of ChatGPT’s role in improving health literacy.

Cohort B: Role in supporting clinical practice

Multiple studies have tested the ability of ChatGPT to pass exams designed for healthcare professionals. A recent study evaluated the ability of ChatGPT to answer questions asked by the American Heart Association Basic Life Support and Advanced Cardiovascular Life Support exams, which healthcare workers often take. ChatGPT was asked to respond to 96 questions. The authors found that while the database did not achieve passing scores for either exam, each answer provided by the platform included detailed explanations with appropriate references to the American College of Cardiology and American Heart Association websites [17]. These results suggest the potential applications of ChatGPT in supporting the education of health professionals, professionals in training, and the general population, but also the importance of caution in the provided answers.

Similarly, Kusunose et al. (2023) tested the ability of ChatGPT to answer clinical questions written in the Japanese Society Hypertension Guidelines. ChatGPT was asked a total of 31 questions. On review of the results, the platform had an overall 64.5% accuracy rate (20/31). The greatest accuracy was demonstrated for evidence-based clinical questions, and the lowest accuracy was seen for questions with limited evidence in the literature. The study also evaluated the likelihood that the platform would give the same answer if asked the same question multiple times by asking each question 10 times. They observed that ChatGPT’s answers were identical for only nine of the 31 questions, indicating the potential for variability [19]. Another study evaluated the ability of ChatGPT to pass the European Cardiology exam. The study assessed a total of 362 sample questions from multiple study resources. The platform achieved an accuracy of 58.5%. The exam’s pass rate is usually 60%, suggesting that ChatGPT appears to be able to achieve scores on par with or above the level of experienced medical professionals [18].

Another group of researchers analyzed ChatGPT’s response regarding its future role in cardiac computed tomography (CT). Questions asked included debate questions adapted from the Society of Cardiovascular CT program. The authors asked questions such as, “What is the value of high-risk plaque assessment on coronary CT to predict patient outcomes?”, “Can coronary CTA quantitative plaque analysis be used to guide patient management?”, and "How will AI transform cardiovascular CT?”. A total of six debate questions were asked. The authors observed that the answers provided appeared plausible and included details supporting both sides of each debate question. However, while appropriate, the responses were also very general and contained no supporting references, hindering the assessment of the validity of the answers [20].

ChatGPT’s ability to rapidly search information and respond to customized prompts raises interest in its potential ability to support direct patient care. An exploratory study asked ChatGPT to provide treatment and management plans for 10 detailed clinical case presentations for common cardiovascular conditions. For example, the platform was asked the following: “A 30-year-old male, presented to a medicine outpatient department with a past medical history of dental infection, pan systolic murmur, digital clubbing, Osler’s nodes, Janeway lesions and petechial rash. What is the diagnosis and treatment?” Other cases included patient presentations of angina pectoris, aortic dissection, mitral regurgitation, mitral stenosis, atrial fibrillation, and Marfan’s syndrome. Cardiologists and medical specialists with cardiovascular care experience critiqued the responses provided by the platform. The platform accurately diagnosed eight of the 10 clinical scenarios. Notably, while the platform misdiagnosed two of these clinical scenarios, the responses provided were not entirely incorrect, as often the diagnosis provided was associated with the actual diagnosis. In the first case, mitral stenosis was misdiagnosed as pulmonary hypertension, and in the second case, mitral regurgitation was diagnosed as dilated cardiomyopathy. However, while responses to treatment and management appeared to be accurate, they lacked specificity [21].

The language model may also be used to support healthcare professional training and help clinicians answer clinical questions. Gala et al. (2023) reported that ChatGPT may support healthcare providers by prompting them to ask appropriate questions to patients. The paper showcases ChatGPT’s response to the following questions, "Patient presents with shortness of breath and orthopnea. What should I ask in the history?” and “Patient presents with shortness of breath and orthopnea. What are some red flag symptoms?”. Additionally, clinicians could ask ChatGPT for support with developing treatment plans for patients. The narrative review showcases ChatGPT's answer to "Patient presents with shortness of breath and orthopnea. I suspect heart failure. What medications should I prescribe?”. The platform’s response includes several medications used to treat heart failure but also provides a cautionary statement stating, “It is important to note that the appropriate medication and dosage will depend on the patient’s individual medical history and the severity of their heart failure” [16].

Another application observed is the ability of ChatGPT to develop simulation cases. Clinicians may input prompts into ChatGPT to create simulation case presentations with appropriate follow-up questions for different levels of training. The authors of an article asked ChatGPT to "Create a simulation case presentation for a patient with heart failure and generate one question for the following levels of training: medical student, resident, and cardiology fellow" and provide answers [16]. In response, the platform was able to appropriately develop clinical scenarios and questions for different levels of training, highlighting a potential application to support clinician education.

These studies highlight ChatGPT’s application in answering clinical questions and supporting medical decision-making for uncomplicated cases. Future providers may use ChatGPT to answer clinical questions alongside existing databases. While ChatGPT successfully answers clinical exam questions, this may not always translate to real-life scenarios. ChatGPT may serve as a resource, but practitioners still need to apply critical thinking when treating patients. More research is necessary to assess ChatGPT’s risks and limitations.

Cohort C & D: Role in research papers, literature reviews, and case reports

Research studies are key for advancing medical knowledge as they enable clinicians to share information, discuss rare diseases, and describe unusual outcomes. The time required for manuscript writing can be a potential barrier hindering academic productivity. Using AI-based tools has the potential to allow researchers to write more efficiently.

The use of ChatGPT to write research articles, literature reviews, and case reports was noted in multiple studies. To assess the platform’s potential, researchers utilized ChatGPT to assist them while conducting an initial literature review on virtual reality (VR) use in cardiology. ChatGPT was tasked with classifying research papers through their abstracts, either as ones that discussed the use of VR use by providers or VR use by patients. For each of the 170 publications, the following prompt was given to ChatGPT, “Can you classify the research into type A or type B based on the following abstract, where type A involves the use of virtual reality devices by healthcare providers and type B involves the use of VR devices by patients? Just answer by type A or B.” The same studies were classified by human researchers. They found that ChatGPT only misclassified four publications out of 170. The platform was able to achieve a sensitivity of 0.98 and a specificity of 0.96 [24].

Similarly, in another literature review paper, the authors successfully used ChatGPT to support each step of the review process. Responses generated by ChatGPT were compared to those by reviewers for each step of the literature review process. ChatGPT was used to screen articles by title and abstract, create a data extraction using patient/population, intervention, comparison, and outcomes (PICO) framework, summarize studies, and assess bias. The authors commented that the platform sometimes had difficulty with the initial literature screening process and assessing bias. The abstract, methods, results, and discussion were also written by ChatGPT in the final published paper. In areas where ChatGPT was not as helpful, the reviewers commented that they were able to adjust their prompt questions to achieve their desired outcome [25]. These studies demonstrate the potential that ChatGPT brings with its near-instantaneous ability to examine literature, especially when compared to the many hours required to read and analyze papers the traditional way.

Marchandot et al. employed ChatGPT prompts to write a paper published in the European Society of Cardiology Journal. The authors provided the platform with relevant information and a series of prompts to generate text that was reviewed and edited to ensure coherence. Prompts used included the following: “Write an article about the pros and cons of ChatGPT in the field of academic research”, “What about ChatGPT for the revision of scientific manuscript”, and “Make a criticism on ChatGPT and ethical concerns in the field of academic research” [23]. Multiple case reports in this literature review also used ChatGPT. Some cases used the platform to write their full case report by submitting a de-identified case and prompting the platform to generate their report's title, introduction, case, and discussion sections. While others submitted their introduction, discussion, and conclusions to the platform for editing.

However, the ethics of using ChatGPT to write publications may be a gray area. Bart et al. (2023) wrote an editorial examining how to use ChatGPT to write papers ethically. The authors reviewed ICMJE guidelines for authors and concluded that the ChatGPT platform likely does not meet all the required author criteria for papers. Additionally, the authors concluded that all uses of ChatGPT or AI technology should be disclosed before manuscripts are submitted, and details should be given on the specific way the platform is used. This transparency is necessary to ensure the integrity of the collected data. Elsevier (Amsterdam, Netherlands), one of the largest publishers of scientific journals, has also released guidelines regarding the use of ChatGPT in response to its increased citation as an author. Elsevier only allows ChatGPT and other AI technologies to be used to improve the readability of a paper. The publication platform also does not allow any AI platform or software to be listed as an author and requires authors to disclose any use of AI in their manuscripts [26].

Limitations of ChatGPT noted in multiple papers are the inability of the platform to access information after 2021 and inaccuracies that sometimes result when the platform provides references for information. In Moons et al., for example, when asked a clinical question, "What is known about the psychological impact of COVID-19 in adults with congenital heart disease?”, the platform responded successfully. However, when prompted to include references to scientific articles, the platform instead provided suggestions for keywords to be searched in PubMed, Scopus, and the Web of Science [22]. Some authors of the case reports included in this literature review noted the limitations of using this platform to write their publications. They observed that ChatGPT sometimes provided inaccurate reference citations or cited nonexistent studies, requiring additional author verification of references [27,29,31,32,34]. Other authors observed that if prompts were not specific, the platform sometimes responded with factually inaccurate or irrelevant information [33,34]. The potential deficits presented in this section emphasize that while ChatGPT has valuable applications in academic literature, researchers should still exercise caution when using it.

Limitations

This literature review has unique limitations given the task of searching large databases. The articles we included were published up until September 2023 to allow for time to analyze the papers. Some papers that may not have been indexed in PubMed at the time of the literature search may have been missed. Additionally, pre-printed papers and those written in other languages were also excluded.

## Conclusions

The increasing popularity of ChatGPT treads unknown territory in medicine. There are many promising applications of the technology, especially within cardiology. ChatGPT can be used to advance health literacy by making complex medical information more accessible and serve as a resource to providers in solving clinical problems. The AI platform may also streamline the process of writing scientific literature through its ability to gather and analyze information rapidly. Further research is needed to better understand the strengths and limitations of ChatGPT in cardiology.
